# Occupational and Leisure-Time Physical Activity Related to Job Stress and Job Satisfaction: Correspondence Analysis on a Population-Based Study

**DOI:** 10.3390/ijerph182111220

**Published:** 2021-10-26

**Authors:** Domingo de-Pedro-Jiménez, Alfonso Meneses-Monroy, Rocío de Diego-Cordero, Marta María Hernández-Martín, Antonio Gabriel Moreno-Pimentel, Manuel Romero-Saldaña

**Affiliations:** 1 University of Cádiz, 11003 Cádiz, Spain; dodepeji@gmail.com; 2 Department of Nursing, Complutense University of Madrid, 28040 Madrid, Spain; martamah@ucm.es (M.M.H.-M.); Antomo05@ucm.es (A.G.M.-P.); 3 Research Group PAIDI-CTS 969 Innovation in HealthCare and Social Determinants of Health, Faculty of Nursing, Physiotherapy and Podiatry, University of Seville, 41009 Seville, Spain; rdediego2@us.es; 4 Grupo Asociado de Investigación GA16 “Estilos de Vida, Innovación y Salud”, Department of Nursing, Pharmacology and Physiotherapy, Instituto Maimónides de Investigación Biomédica de Córdoba (IMIBIC), University of Córdoba, 14004 Córdoba, Spain; z92rosam@uco.es

**Keywords:** occupational health, job stress, job satisfaction, exercise, sex distribution, leisure-time physical activity

## Abstract

Background: Leisure-time physical activity (LTPA) is not the same as occupational activity. Various factors influence both forms of physical activity, including job stress and job satisfaction, but the associations found are weak, and the need for new studies in large populations is emphasized. The objective was to study the relationship between job stress and job satisfaction, and the relationship between these and occupational and leisure-time physical activity according to the National Survey of Health 2017. Methods: A population-based study of 8716 workers between 18 and 65 years of age. The variables age, sex, leisure, and occupational-time physical activity (OTPA), educational level, type of occupation, job stress level, and job satisfaction were collected. A simple and multiple correspondence analysis was performed between the variables that reached statistical significance. Results: 4621 cases (53.02%) correspond to men with a mean age of 44.83 years (SD 10.22) and 4095 cases to women with a mean age of 44.55 years (SD 10.23). Women had higher percentages of higher education (*p* < 0.001), intermediate to high occupations and unskilled (*p* < 0.001), job stress (*p* < 0.001), covered the most extreme levels of satisfaction (*p* = 0.003), and do less LTPA (*p* < 0.001) and OTPA (*p* < 0.001). Also, in women a relationship was found between job stress and LTPA (*p* = 0.024), as well as between satisfaction and both forms of physical activity (OTPA *p* = 0.013 and LTPA *p* < 0.001). In men, significance was only reached in the relationship between job stress and OTPA (*p* <0.001). Conclusions. The higher the job stress, the less the job satisfaction, but the relationship is reversed in the intermediate categories. For both sexes, job stress is related to a sedentary lifestyle and higher employment and education levels. Higher levels of satisfaction correspond to higher levels of occupancy. The relationship between job satisfaction and educational level is direct in women but inverse in men. In women, there is a relationship between sedentary occupations and job satisfaction. In addition, intense physical activity at work is related to higher levels of job stress, lower satisfaction levels, and less physical activity in leisure-time.

## 1. Introduction

Physical activity influences well-being, which is based on vital areas such as leisure, work, and health [1]. However, leisure-time physical activity (LTPA) is not the same as occupational-time physical activity (OTPA) [2]. Various socioeconomic, lifestyle, personal, gender, and work factors influence both forms of physical activity, resulting in a different physiological response [3,4]. In turn, they influence each other, although not reciprocally: a better physical condition will result in better performance of work tasks that require effort [5] but making great work effort negatively influences the performance of physical activities of leisure. OTPA increases the risk of absenteeism while LTPA decreases it [6,7]. 

Among the various factors related to physical activity are job stress and job satisfaction. Job satisfaction, a comparison between real work and worker expectations, is by itself a multidimensional factor, dependent on personal, organizational, and environmental conditions [8]. Poor job satisfaction is associated with mental problems, such as depression, anxiety, or burn-out syndrome [9]. The few studies that have studied the relationship between this factor and physical activity have found weak associations, highlighting the need for more studies in this regard [5]. 

Somewhat more abundant are those that relate physical activity and job stress, defined by the International Labour Organization (ILO) as “the physical and emotional response to damage caused by an imbalance between the perceived demands and the perceived resources and capacities of an individual to meet these demands” [10]. High labour demand, effort-reward imbalances, characteristics of the job itself, lack of social support or individual skills could be behind physical inactivity. Some individuals use physical exercise as a tool to manage stress, yet for others it is the stress itself that prevents them from being more physically active [11,12,13]. This bidirectional relationship, the studies focused on specific populations and, in general, scarce, stimulate further investigation on these issues [14,15]. 

The very relationship between job stress and job satisfaction requires further studies [16] as well as inequalities related to age, sex, economic or education, among others [17]. 

Gender inequality is a common risk factor for physical activity, job satisfaction, and job stress. It is present in all occupations worldwide [18] and requires a specific approach to highlight these differences to understand how to reduce them.

For this, the Spanish National Health Survey (ENSE) offers a valuable tool to address all the variables mentioned in a large sample of both sexes and with a high representation of workers of all types. Based on this survey, we set the following objectives: (i) to study the relationship between job stress and job satisfaction; (ii) to study the relationship between the above and OTPA/LTPA.

## 2. Materials and Methods

### 2.1. Study Design Population and Sample

Population-based cross-sectional study carried out from the ENSE 2017 (Ministry of Health, Consumption and Social Welfare with the collaboration of the National Institute of Statistics) [19], which collects health information related to the population resident in Spain in 23,860 households and a sample of 29,800 people. 

In the ENSE, stratified three-stage sampling was carried out, distributing the sample among autonomous communities uniformly and proportional to its size. Likewise, the census sections were selected within each stratum with a probability proportional to its size. In each section, the dwellings were extracted by systematic sampling after sorting by size, which led to self-weighted samples in each stratum. Kish’s random procedure was used to select people, which assigns equal probability to all household members. 

Concerning the eligibility criteria, subjects aged between 18 and 65 years and working at the time of the ENSE were included in the sample. By contrast, the cases that answered “does not know” or “does not answer” of any of the analyzed variables were excluded, as well as the values that did not belong to any of the items collected in the scales. 

The final sample consisted of 8716 workers aged between 18 and 65 years. 

### 2.2. Variables and Measurements

Sociodemographics and habits: age, sex, physical activity in leisure-time, education level.

Labour: occupation, job stress level, job satisfaction level, physical activity at work. 

Data on physical activity were specifically collected using the short version of the International Physical Activity Questionnaire (IPAQ). Validated for the Spanish population [20,21], it has shown a reliability of 0.65 (r = 0.76; 95% CI = 0.73–0.77). The validity coefficients suggest that the long and short versions have acceptable reliability (r = 0.67; 95% CI = 0.64–0.70).

The variables’ job stress level and level of job satisfaction were divided into seven categories that the ENSE identified from no stress or satisfaction to extremely stressful or satisfactory, without naming the intermediate categories. All categories have been renamed for easy identification. These and the rest of the categories of the other variables can be consulted in Table 1. 

### 2.3. Ethical and Legal Aspects 

The use of ENSE data does not require approval by an ethics committee. Files for public use are not considered confidential according to Regulation (EU) 2016/679.

### 2.4. Statistic Analysis 

The IBM SPSS Statistics 24.0 software (IBM, Chicago, IL, USA) was used for statistical analysis. The quantitative variables have been represented by the arithmetic mean and the standard deviation, while the qualitative variables were summarized according to their absolute frequencies and percentages. 

The comparison of arithmetic means was carried out using the one-way ANOVA test. 

For the analysis of contingency tables with polytomous nominal and dichotomous variables, the chi-square statistical contrast test and Fisher’s test were used, and the Cochran-Armitage test for dichotomous and ordinal variables. 

Somers’ d test was carried out to determine a linear association between ordinal variables, considered dependent, and nominal and ordinal variables. 

Kendall’s tau b correlation was used to evaluate the relationship between ordinal variables. Simple correspondence analysis was performed and a multiple correspondence analysis between the variables that were significant in the unifactorial analysis according to sex.

## 3. Results

### 3.1. Characteristics of the Study Sample

Table 1 shows the percentages of each variable according to sex. Except for age, in the rest of the variables, the differences between the sexes were significant. 

Of the 8716 selected cases, 4621 (53.02%) were men with a mean age of 44.83 years (SD = 10.22), and 4095 (46.98%) were women with a mean age of 44.55 years (SD = 10.23).

Higher education levels, from intermediate vocational education to university studies, predominated among women. 

Regarding occupations, the intermediate to high (managing directors of companies) were higher in women and the category of unskilled workers. Men were more abundant in the categories of supervisors and skilled technical occupations and skilled workers in the primary sector and other semi-skilled sectors. 

LTPA reached higher percentages in men, in the categories of several times a month and several times a week, while women were more abundant in the categories none or occasionally.

The highest job stress levels predominated in women. In terms of the level of job satisfaction, nothing and almost nothing satisfactory and highly satisfactory, the extremes were also higher in women. 

### 3.2. Univariate Inferential Analysis

Table 2 shows the univariate inferential analysis. The relationship between job stress and job satisfaction level and between job stress and occupation and educational level reached high significance in both sexes. Women also appeared to show LTPA associated with job stress (*p* = 0.024). In the relationship between job satisfaction and occupation and educational level, both sexes also reached significance, and women, again, with LTPA (*p* = 0.001) and OTPA (*p* = 0.013). It should be noted that significant differences were found between the sexes in the relationship between job satisfaction and educational level.

**Figure 1:**
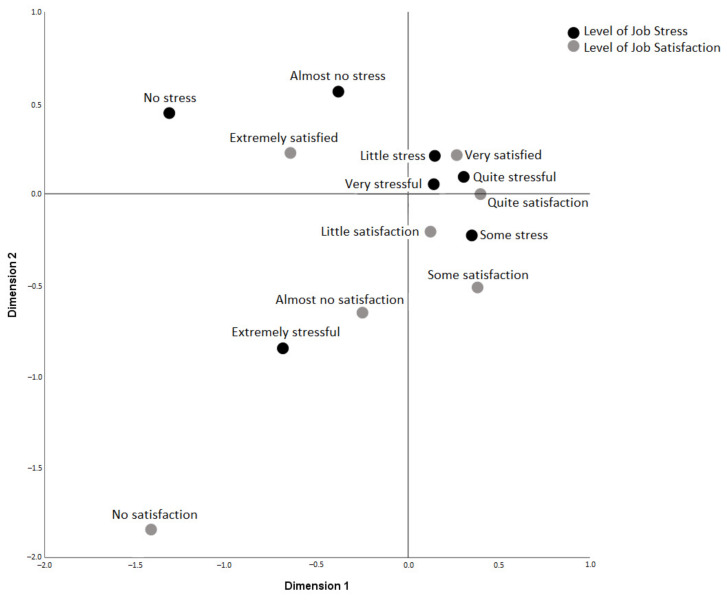
Simple correspondence analysis between job stress level and job satisfaction.

Figure 1 shows the simple correspondence analysis graph between job stress and job satisfaction (83% accumulated inertia, *p* < 0.001). In dimension 1, both for stress and satisfaction, extreme values are shown (not at all, almost nothing and extremely stressful or satisfactory) compared to average values (little, somewhat, very, quite). In dimension 2, extremely and somewhat stressful values are opposed to almost nothing, tiny, very, and relatively stressful. Job satisfaction values are not at all; almost nothing, minor, or somewhat are opposed to fairly, very, and extremely satisfactory values. Among variables, the relationship between low levels of job stress (nothing and almost nothing) with extremely high levels of satisfaction is observed and the extreme level of job stress with low levels (nothing or almost nothing) of job satisfaction. 

**Figure 2:**
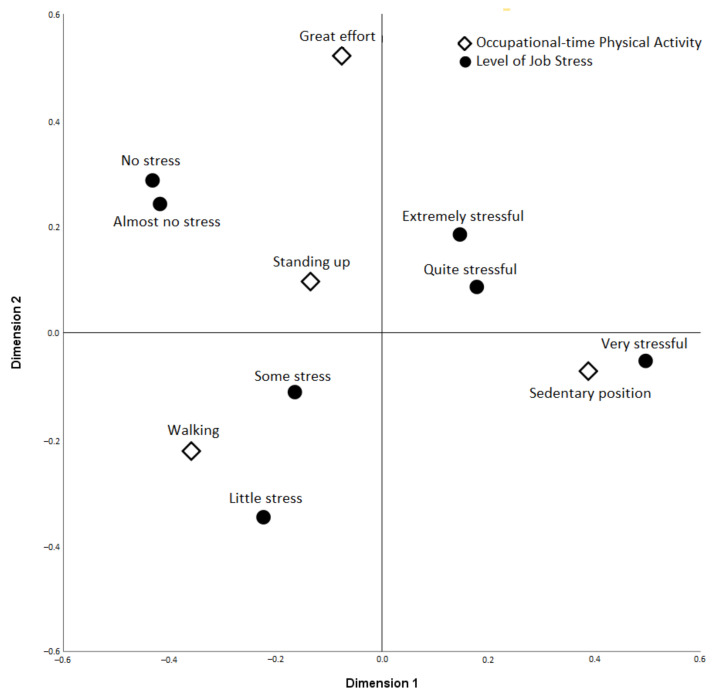
Simple correspondence analysis between job stress level and occupational physical activity (men).

The unifactorial analysis in men only found a relationship between job stress and OTPA, so a simple correspondence analysis was performed between both variables, shown in Figure 2. With cumulative inertia of 88.7% (*p* < 0.001), it can be observed that in dimension 1 the categories walking, standing and making great efforts are opposed to sedentary positions and the levels quite, very and extremely stressful. Dimension 2 groups the categories of walking and sedentary positions versus standing and making great efforts, and in terms of stress, the levels of a minor, some and very stressful are observed compared to the categories of nothing, almost nothing, a lot and extremely stressful. The relationships between the categories of both variables show that sedentary positions are more directly related to the very stressful level and walking with minor or somewhat stressful levels. Standing without making great movements or efforts or performing tasks that require significant effort are less direct relationships.

### 3.3. Multiple Correspondence Analysis

In women, the analysis gave greater weight in dimension 1 to the level of job stress (0.501), level of satisfaction (0.439) and OTPA (0.292). In dimension 2, similar values were obtained in the same order: job stress level (0.462), satisfaction level (0.436) and OTPA (0.219). The two dimensions together explained 63.9% of the variability. 

**Figure 3:**
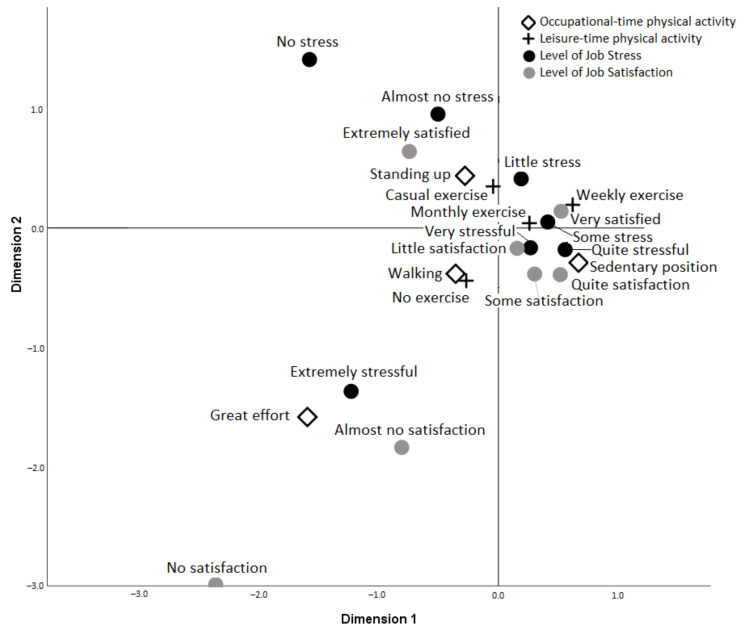
Multiple correspondence analysis (women).

Figure 3 shows that dimension 1 contains the same categories of job stress and job satisfaction (not at all, almost at all, and extreme) versus little, somewhat, significantly, and quite a bit. The categories of exerting effort, walking, or standing are opposed to being sedentary.

Moreover, do not exercise leisurely or do it occasionally was compared to weekly or monthly exercise. Dimension 2 contains the categories quite, very and extremely stressful versus somewhat stressful, little, almost nothing, and nothing. Satisfaction levels were not at all, almost at all, little, somewhat, and quite satisfactory versus very and extremely satisfactory. Exerting great efforts in the workplace, walking or being sedentary are opposed to standing at work. Not doing physical activity in your spare time faces doing it on an occasional, weekly, or monthly basis. In the relationships between variables, it is observed that hard work physical activity is more directly related to extreme levels of job stress and low levels of job satisfaction (almost nothing). In addition, the category that does not perform LTPA is the one closest to great efforts in the workplace.

On the other hand, sedentary positions are closer to the fairly and very stressful levels and the somewhat and quite satisfactory categories. Performing monthly to weekly exercise is more closely related to sedentary positions.

Walking at work and not doing LTPA are closely related. Standing at work and occasional exercise are also related.

## 4. Discussion

A cross-sectional descriptive study was carried out on the Spanish population of working age to study the relationship between job stress and job satisfaction and analyze the relationship between these and OTPA/LTPA. 

The main results suggest that job satisfaction decreases the more job stress is perceived, although this relationship is reversed when the levels are not extreme. The higher the sedentary lifestyle at work, the level of occupation and studies, the higher the job stress levels in both sexes. The relationship between job satisfaction and educational level is direct in women but inverse in men. Moreover, in women, intense physical activity at work is related to higher job stress levels, lower job satisfaction levels, and less LTPA. 

As previous studies have already pointed out [16,22], we found a significant relationship between job stress and job satisfaction, very evident in the extreme categories of stress, as shown by the simple correspondence analysis. It is also interesting to observe how the intermediate categories are related to each other. The somewhat stressful category is equidistant from the little, somewhat, and fairly satisfying levels. The fairly stressful category is also equidistant from being fairly or very satisfied. The differentiation between distress and eustress may be behind these results [23]. High levels of stress correspond to negative stress (distress) and, therefore, not at all or almost not at all satisfactory, and medium to low levels correspond to positive stress (eustress), which is closer to the highest levels of satisfaction. 

We find that job stress and job satisfaction are higher as the level of occupation increases in both sexes. The inverse relationship between stress and satisfaction mentioned above could lead us to think it contradicts this finding. However, dissatisfaction related to job stress is not the same as dissatisfaction due to occupational level. The relationship between high levels of occupation and job satisfaction is also influenced by many factors, among which organizational factors, including remuneration, promotion, and main motivation have greater weight [8]. 

We also found a relationship between the education level and job stress in both sexes, in such a way that its levels increase as the education level is higher. This is in line with the relationship between job stress and occupation level since we verify the relationship between higher levels of occupation and higher levels of studies, which reached a high significance (*p* < 0.001) in both sexes. 

The relationship between job satisfaction and educational level also reached significant values. However, the direction of the relationship was opposite according to sex. Job satisfaction increases as the studies are higher in women, but the opposite occurs in men. This difference has been revealed in other studies [24,25] and has been considered a paradox since, although women have worse working conditions in terms of labour and salary segregation, they tend to be more satisfied with their jobs [26]. The higher concentration of women in the group of higher studies (from intermediate to university degrees) and men in the lower studies groups could interfere in this relationship. In the Spanish population, it has been found that the differences are more minor in groups with a higher educational level [27], but this does not explain the paradox. The greater difficulties of access to the labour market for women could, on the other hand, originate in higher levels of satisfaction than men due to the fact that they have been able to access it. The differences between the types of working day could also be influencing this. Women are the majority when it comes to short hours, possibly influenced by the type of work they are required to do or by the need to combine work and family responsibilities [18,28]. How each sex perceives each level of satisfaction would be an issue to consider; and different perceptions may be taking place that stimulate future research [27].

Concerning physical activity at work by men, we only found a relationship with job stress, in such a way that higher stress levels are associated with more sedentary work activity. Simple correspondence analysis shows this relationship. The categories quite and mainly very stressful and sedentary position are very close, as well as the categories walk (carrying some weight, making frequent movements) together with little or some stress. The relationship between sedentary positions and high stress is associated with higher occupations, in line with the previous reasoning between occupation and job stress. These positions are related to tasks where intellectual function predominates and requires little or no physical effort [29]. Staying on your feet most of the day without making large movements or efforts is almost equidistant from the categories, almost nothing, something, quite a bit and extremely stressful. The breadth of working conditions could explain this variability that standing can encompass, from an operator on an assembly line with high pace requirements and scheduled breaks to shop assistants in low-load situations, for example. Although the beneficial effects of physical activity are almost exclusively associated with leisure time and exercise with specific characteristics versus physical activity at work, expressed in various studies as a paradox [30,31], jobs that allow moving without making efforts could have positive effects from psychological points of view. For example, this could be by increasing the possibility of intra-work interaction and social support, an anti-stress factor mentioned by other authors [32]. This is another issue that can stimulate future research. 

The lack of a relationship in men between LTPA and job stress could be due to the lower presence of men in sedentary jobs and the fact that men exercise more than women. There are studies that predict behavioural changes in either direction of stress, i.e., LTPA reduces the effects of stress but also stress prevents being more physically active [11,12]. It seems more plausible to consider that it is the sum of less exposure to job stress and more LTPA that may be behind our results.

In women, we did not find a relationship between job stress and OTPA, but we did find a relationship with LTPA. The negative sign of the relationship tells us that the less physical activity, the more job stress. The relationship between LTPA and absenteeism reduction has been confirmed [7] and precisely the effect on job stress [33]. 

Regarding satisfaction, unlike in men, we found a positive relationship for both types of physical activity, in such a way that higher levels of satisfaction were associated with more significant physical work requirements and greater physical and sports activities in free time. The greater satisfaction with more significant physical labour requirements is conditioned by the greater number of women in the groups with fewer work efforts and must be evaluated in conjunction with the other related variables. The multiple correspondence analysis, which shows the relationships between the categories of the four variables, confirms the relationship between high job stress and low job satisfaction and exertion at work and not exercising in leisure time. In addition to the bidirectional relationship between stress and physical activity, the explanation could lie in the high-energy demand at work, which would prevent new physical activities and because jobs that require great effort are mostly associated with low socioeconomic and educational conditions [34]. In line with this statement is that the categories of greater LTPA in sedentary positions are associated since they have less energy expenditure during work activity and better socioeconomic and educational conditions.

Following the same reasoning as for women, the lack of association between job satisfaction and both types of physical activity may be due to the higher presence of men in the moderate levels of satisfaction and in the groups with higher workplace effort, lower qualification and education levels. However, these gender differences are multidimensional in origin. We have found significant differences in all the variables analyzed (except for age, probably due to the restriction to workers between 18 and 65 years old). Occupational and educational differences can already condition these differences with respect to job stress, job satisfaction, and physical activity, but also differences in stress management between the sexes, perception of health, roles at home and in the workplace, or socioeconomic differences due to gender discrimination [10,24,35]. 

The study limitations are diverse. To those related to the type of study, we must add how the information is collected and the absence of assessment of all the variables involved. 

As a strength, it is worth highlighting the randomization process used in the survey, and the large sample size analyzed, which offers guarantees of its external validity, extendable in our case to the Spanish working population. 

The evolution of personal and social needs throughout life can influence our levels of satisfaction. Longitudinal studies that assess these aspects are necessary, but they can be highly costly from all points of view, so a systematic approach to the problem addressed is essential.

## 5. Conclusions

Responding to the proposed objective, we find that there is a manifest inverse relationship between the extreme categories of job stress level and job satisfaction—the higher the stress, the less satisfaction—but the relationship becomes direct in the intermediate categories. 

For both sexes, higher levels of job stress correspond to higher levels of occupation and studies. Also, in both sexes, higher levels of satisfaction correspond to higher levels of employment. 

The relationship between job satisfaction and educational level is direct in women, higher levels of satisfaction correspond to higher educational levels but are inverse in men. 

In men, the only relationship between physical activity, at work or leisure, and job stress or job satisfaction, was observed more clearly between sedentary positions and higher stress levels. 

Also, there is a relationship between a sedentary lifestyle and higher levels of job stress and job satisfaction in women. In addition, intense physical activity at work is related to higher job stress levels, lower job satisfaction levels, and less physical activity in leisure-time.

Occupational health services should periodically evaluate the job stress level of workers with higher levels of occupation, mainly among those with sedentary jobs.

They should also pay special attention to workers who perform intense physical efforts, in addition to being more stressed and dissatisfied with their jobs, which limits them in terms of one of the most effective tools to combat these risk factors, such as LTPA. This fact could make them more susceptible to accidents and occupational diseases.

Our study reveals important gender differences. The direct relationship between job satisfaction and level of education among women and the inverse among men should be studied in greater depth, assessing all the influencing factors, including social and work-related cultural factors.

## Figures and Tables

**Table 1:** Characteristics of the sample according to sex variable (*n*= 8716).

		Men (*n* = 4621)	Women (*n* = 4095)	*p* Value
Age		44.83 (SD = 10.22)	44.55 (SD = 10.23)	0.431
Job stress level	No stress	295 (6.4%)	281 (6.9%)	0.001
	Almost no stress	420 (9.1%)	323 (7.9%)	
	Little stress	630 (13.6%)	506 (12.4%)	
	Some stress	999 (21.6%)	807 (19.7%)	
	Quite stressful	1171 (25.3%)	1078 (26.3%)	
	Very stressful	675 (14.6%)	621 (15.2%)	
	Extremely stressful	431 (9.3%)	479 (11.7%)	
Job satisfaction level	No satisfaction	81 (1.8%)	79 (1.9%)	0.003
	Almost no satisfaction	97 (2.1%)	97 (2.4%)	
	Little satisfaction	245 (5.3%)	185 (4.5%)	
	Some satisfaction	595 (12.9%)	495 (12.1%)	
	Quite satisfaction	1071 (23.2%)	849 (20.7%)	
	Very satisfied	1312 (28.4%)	1125 (27.5%)	
	Extremely satisfied	1220 (26.4%)	1265 (30.9%)	
Occupation	Managing director of companies with 10 or more employees and professionals associated with university degrees	629 (13.6%)	665 (16.2%)	<0.001
	Managing director of companies with less than 10 employees and professionals associated with university diploma. Athletes and artists.	448 (9.7%)	469 (11.5%)	
	Intermediate occupations and self-employed workers	985 (21.3%)	975 (23.8%)	
	Supervisors and workers in qualified technical occupations	755 (16.3%)	395 (9.6%)	
	Skilled workers in the primary sector and other semi-skilled workers	1393 (30.1%)	1131 (27.6%)	
	Unskilled workers	411 (8.9%)	460 (11.2%)	
Education level	He does not know to read nor to write	2 (0.04%)	4 (0.1%)	<0.001
	Incomplete Primary Education (<5 years in school)	78 (1.7%)	36 (0.9%)	
	Complete Primary Education.	384 (8.3%)	246 (6.0%)	
	Elementary Bachelor	1328 (28.7%)	816 (19.9%)	
	Bachelor	606 (13.1%)	522 (12.7%)	
	Professional education of intermediate degree or equivalent	435 (9.4%)	425 (10.4%)	
	Professional education of a higher degree or equivalent	647 (14.0%)	503 (12.3%)	
	University studies or equivalent	1141 (24.7%)	1543 (37.7%)	
Occupational-time physical activity	Sitting most of the day	1579 (34.2%)	1430 (34.9%)	<0.001
	Standing for most of the day without making great movements or efforts	1719 (37.2%)	1935 (47.3%)	
	Walking. Carrying some weight.	995 (21.5%)	604 (14.7%)	
	Performing tasks that require great physical effort	328 (7.1%)	126 (3.1%)	
Leisure-time physical activity	None	1467 (31.7%)	1490 (36.4%)	<0.001
	Occasionally	1434 (31.0%)	1531 (37.4%)	
	Sometimes in the month	898 (19.4%)	548 (13.4%)	
	Several times in a week	822 (17.8%)	526 (12.8%)	

**Table 2:** Univariate analysis according to sex (*n* = 8716).

		Men (*n* = 4621)		Women (*n* = 4095)	
		Indicator	*p*	Indicator	*p*
Job stress	Job satisfaction	Ʈb = −0.076	<0.001	Ʈb = −0.084	<0.001
	Occupation	d = −0.1	<0.001	D = −0.047	<0.001
	Education level	d = 0.068	<0.001	d = 0.055	<0.001
	Occupational-time Physical Activity	d = −0.063	<0.001	d = −0.017	0.24
	Leisure-time Physical Activity	Ʈb = −0.005	0.666	Ʈb = −0.029	0.024
Job satisfaction	Occupation	D = −0.047	<0.001	D = −0.075	<0.001
	Education level	d = −0.025	0.031	d = 0.055	<0.001
	Occupational-time Physical Activity	d = 0.019	0.136	d = 0.037	0.013
	Leisure-time Physical Activity	Ʈb = 0.005	0.681	Ʈb = 0.043	0.001

## Data Availability

The data supporting the results presented can be obtained from the website of the Spanish National Statistics Institute (INE). http://www.ine.es (accessed on 14 January 2021).

## References

[1] Lera-López F., Ollo-López A., Sánchez-Santos J.M. (2016). How Does Physical Activity Make You Feel Better? The Mediational Role of Perceived Health. Appl. Res. Qual. Life.

[2] Harari G., Green M.S., Zelber-Sagi S. (2015). Combined association of occupational and leisure-time physical activity with all-cause and coronary heart disease mortality among a cohort of men followed-up for 22 years. Occup. Environ. Med..

[3] Holtermann A., Krause N., Van Der Beek A.J., Straker L. (2017). The physical activity paradox: Six reasons why occupational physical activity (OPA) does not confer the cardiovascular health benefits that leisure time physical activity does. Br. J. Sports Med..

[4] The Lancet Public Health (2019). Time to tackle the physical activity gender gap. Lancet Public Health.

[5] Da Rocha Morgado F.F., de Souza do Vale W., Lopes C.S., de Albuquerque Maranhão Neto G., Lattari E., Mediano M.F.F., Rostila M., Griep R.H., Machado S., Penna T.A. (2020). Psychosocial determinants of physical activity among workers: An integrative review. Rev. Bras. Med. Trab..

[6] Holtermann A., Hansen J.V., Burr H., Søgaard K., Sjøgaard G. (2011). The health paradox of occupational and leisure-time physical activity. Br. J. Sports Med..

[7] Kerner I., Rakovac M., Lazinica B. (2017). Leisure-time physical activity and absenteeism. Arch. Ind. Hyg. Toxicol..

[8] Sánchez-Sellero M.C., Sánchez-Sellero P., Cruz-González M.M., Sánchez-Sellero F.J. (2014). Organizational characteristics in the labour satisfaction in Spain. Rev. Adm. Empres..

[9] Andersen L.L., Fishwick D., Robinson E., Wiezer N.M., Mockałło Z., Grosjean V. (2017). Job satisfaction is more than a fruit basket, health checks and free exercise: Cross-sectional study among 10,000 wage earners. Scand. J. Public Health.

[10] Organización Internacional del Trabajo (2016). Estrés en el Trabajo. Un Reto Colectivo. [Stress at Work. A Collective Challenge].

[11] Stults-Kolehmainen M.A., Sinha R. (2014). The effects of stress on physical activity and exercise. Sport Med..

[12] Häusser J., Mojzisch A. (2017). The physical activity-mediated Demand-Control (pamDC) model: Linking work characteristics, leisure time physical activity, and well-being. Work Stress.

[13] Fransson E.I., Heikkilä K., Nyberg S.T., Zins M., Westerlund H., Westerholm P., Väänänen A., Virtanen M., Vahtera J., Theorell T. (2012). Job strain as a risk factor for leisure-time physical inactivity: An individual-participant meta-analysis of up to 170,000 men and women. Am. J. Epidemiol..

[14] Buss J. (2012). Associations between Obesity and Stress and Shift Work among Nurses. Workplace Health Saf..

[15] Nyberg S.T., Heikkilä K., Fransson E., Alfredsson L., De Bacquer D., Bjorner J.B., Bonenfant S., Borritz M., Burr H., Casini A. (2012). Job strain in relation to body mass index: Pooled analysis of 160000 adults from 13 cohort studies. J. Intern. Med..

[16] Pujol-Cols L.J., Dabos G.E. (2018). Satisfacción laboral: Una revisión de la literatura acerca de sus principales determinantes. Estud. Gerenciales.

[17] Gell N.M., Wadsworth D.D. (2014). How Do They Do It: Working Women Meeting Physical Activity Recommendations. Am. J. Heal. Behav..

[18] Boniol M., McIsaac M., Xu L., Wuliji T., Diallo K., Campbell J. (2019). WHO|Gender Equity in the Health Workforce: Analysis of 104 Countries [Internet].

[19] Ministerio de Sanidad Servicios Sociales e Igualdad (2017). Encuesta Nacional de Salud. España 2017 (ENSE 2017) [Spanish National Health Survey] [Internet]. Vol. 6..

[20] Pérez_Soto J., García_Cantó E. (2012). Medición de la actividad física mediante el International Physical Activity Questionnaire (IPAQ) en estudios españoles e internacionales [Physical Activity measurement through the International Physical Activity Questionnaire (Ipaq) in Spanish and International studies]. Act. Física Cienc..

[21] Mantilla Toloza S., Gómez-Conesa A. (2007). El cuestionario internacional de actividad física. Un instrument adecuado en el seguimiento de la actividad física poblacional [International Physical Activity Questionnaire. An adequate instrument in population physical activity monitoring]. Rev. Iberoam. Fisioter. Kinesiol..

[22] Estefanía Osorio J., Cárdenas Niño L. (2017). Estrés laboral: Estudio de revisión [Work stress: A review study]. Divers. Perspect. Psicol..

[23] Vidal Lacosta V. (2019). El Estrés Laboral. Análisis y Prevención [Occupational Stress: Analysis and Prevention].

[24] Sánchez Cañizares S.M., Fuentes García F.J., Artacho Ruiz C. (2007). La satisfacción laboral desde la perspectiva de género: Un análisis empírico mediante modelos logit y probit [Job satisfaction from a gender perspective: An empirical analysis using logit and probit models]. Conoc. Innov. Emprend. Camino Futur..

[25] Hauret L., Williams D.R. (2017). Cross-National Analysis of Gender Differences in Job Satisfaction. Ind. Relat..

[26] Fernandez Puente A., Sanchez-Sanchez N. (2020). Once in the Public Sector, Do Differences in Job Satisfaction by Gender Disappear?. Hacienda Publica Esp..

[27] Sánchez-Sánchez N., Fernández Puente A.C. (2019). Is women’s job satisfaction higher than men´s? Self-selection, expectations or utility function. Acta Oeconomica.

[28] OSALAN-Instituto Vasco de Seguridad y Salud (2021). Principales Factores de Género que Inciden en las Actitudes y Comportamientos ante los Riesgos Laborales y en los daños Derivados de los Mismos [Main Gender Factors Affecting Attitudes and Behaviors in Relation to Occupational Hazards and Related Damages].

[29] Dėdelė A., Miškinytė A., Andrušaitytė S., Bartkutė Ž. (2019). Perceived stress among different occupational groups and the interaction with sedentary behaviour. Int. J. Environ. Res. Public Health.

[30] Cillekens B., Lang M., Van Mechelen W., Verhagen E., Huysmans M.A., Holtermann A., Van Der Beek A.J., Coenen P. (2020). How does occupational physical activity influence health? An umbrella review of 23 health outcomes across 158 observational studies. Br. J. Sports Med..

[31] Ferrario M.M., Roncaioli M., Veronesi G., Holtermann A., Clays E., Borchini R., Cavicchiolo M., Grassi G., Cesana G. (2018). Differing associations for sport versus occupational physical activity and cardiovascular risk. Heart.

[32] Navinés R., Martín-Santos R., Olivé V., Valdés M. (2016). Estrés laboral: Implicaciones para la salud física y mental [Work-related stress: Implications for physical and mental health]. Med. Clin..

[33] Azofeifa Mora C. (2018). Revisión de los beneficios de la intensidad y modalidades del ejercicio físico sobre el estrés psicológico [Review of the benefits of physical exercise intensity and modalities on psychological stress]. Pensar Mov. Rev. Cienc. Ejerc. Salud.

[34] Stalsberg R., Pedersen A.V. (2018). Are differences in physical activity across socioeconomic groups associated with choice of physical activity variables to report?. Int. J. Environ. Res. Public Health.

[35] Instituto Nacional de Seguridad y Salud en el Trabajo (INSST) (2018). Condiciones de Trabajo Según Género en España 2015 [Working Conditions by Gender in Spain 2015] [Internet]. https://www.insst.es/documentacion/catalogo-de-publicaciones/condiciones-de-trabajo-segun-genero-en-espana-2015-ano-2018.

